# Importance of Immediate Electronic-Based Feedback to Enhance Feedback for First-Time CPR Trainees

**DOI:** 10.3390/ijerph18083885

**Published:** 2021-04-07

**Authors:** Patrycja Misztal-Okońska, Krzysztof Goniewicz, Mariusz Goniewicz, Jamie Ranse, Attila J. Hertelendy, Lesley Gray, Eric Carlström, Jarle Løwe Sørensen, Amir Khorram-Manesh

**Affiliations:** 1 Department of Emergency Medicine, Medical University of Lublin, 20-059 Lublin, Poland; mariusz.goniewicz@umlub.pl; 2 Department of Aviation Security, Military University of Aviation, 08-521 Dęblin, Poland; k.goniewicz@law.mil.pl; 3 School of Nursing and Midwifery, Griffith University, Gold Coast, QLD 4215, Australia; j.ranse@griffith.edu.au; 4 Department of Emergency Medicine, Gold Coast Health, Gold Coast, QLD 4215, Australia; 5 Fellowship in Disaster Medicine, Department of Emergency Medicine, Beth Israel Deaconess Medical Centre, Boston, MA 02215, USA; attila.hertelendy@georgetown.edu; 6 Department of Emergency Medicine, Harvard Medical School, Boston, MA 02215, USA; 7 Department of Information Systems and Business Analytics, College of Business, Florida International University, Miami, FL 33119, USA; 8 Department of Primary Health Care and General Practice, University of Otago, Wellington 6242, New Zealand; lesley.gray@otago.ac.nz; 9 Joint Centre for Disaster Research, Massey University, Wellington 6021, New Zealand; 10 Gothenburg Emergency Medicine Research Group (GEMREG), Sahlgrenska University Hospital, 413 45 Gothenburg, Sweden; eric.carlstrom@gu.se; 11 Institute of Health and Care Sciences, Sahlgrenska Academy, University of Gothenburg, 405 30 Gothenburg, Sweden; 12 USN School of Business, University of South-Eastern Norway, 3616 Kongsberg, Norway; jarle.sorensen@usn.no; 13 Institute of Clinical Sciences, Department of Surgery, Sahlgrenska Academy, Gothenburg University, 413 45 Gothenburg, Sweden; amir.khorram-manesh@surgery.gu.se; 14 Department of Development and Research, Armed Forces Center for Defense Medicine, 426 76 Gothenburg, Västra Frölunda, Sweden

**Keywords:** first aid, education, basic life support, cardiopulmonary resuscitation, feedback device, simulation, quality, skill retention, motivation, chest recoil, mannequin

## Abstract

Sudden cardiac arrest is one of the leading causes of death globally. The recommended clinical management in out-of-hospital cardiac arrest cases is the immediate initiation of high-quality cardiopulmonary resuscitation (CPR). Training mannequins should be combined with technology that provides students with detailed immediate feedback on the quality of CPR performance. This study aimed to verify the impacts of the type of feedback (basic or detailed) the responders receive from the device while learning CPR and how it influences the quality of their performance and the motivation to improve their skills. The study was conducted at the Medical University of Lublin among 694 multi-professional health students during first aid classes on basic life support (BLS). The students first practiced on an adult mannequin with a basic control panel; afterward, the same mannequin was connected to a laptop, ensuring a detailed record of the performed activities through a projector. Next, the participants expressed their subjective opinion on how the feedback provided during the classes, basic vs. detailed, motivated them to improve the quality of their CPR performance. Additionally, during the classes, the instructor conducted an extended observation of students’ work and behavior. In the students’ opinion, the CPR training with detailed feedback devices provided motivation for learning and improving CPR proficiency than that with a basic control panel. Furthermore, the comments given from devices seemed to be more acceptable to the students, who did not see any bias in the device’s evaluation compared to that of the instructor. Detailed device feedback motivates student health practitioners to learn and improve the overall quality of CPR. The use of mannequins that provide detailed feedback during BLS courses can improve survival in out-of-hospital cardiac arrest.

## 1. Introduction

The immediate initiation of cardiopulmonary resuscitation (CPR) by bystanders in an incident is crucial for the survival of patients in cases of out-of-hospital cardiac arrest (OHCA). OHCA is a major health problem in large parts of the world, with an incidence in Europe of 89/100,000 person-years [1]. Although 30 day survival is greatly improved when cardiopulmonary resuscitation (CPR) is performed before the emergency medical service (EMS) arrives, the death rate is still almost 90% [2,3].

The International Consensus on CPR and Emergency Cardiovascular Care Science with Treatment and Recommendations, developed by the International Liaison Committee on Resuscitation (ILCOR), published updated recommendations and guidelines for CPR in 2020 [4]. The authors highlighted evidence that supports the important relationship between quality of CPR and resuscitation outcomes [4,5]. Two key recommendations from the 2020 ILCOR resuscitation guidelines are the use of real-time audiovisual feedback as a means to maintain CPR quality and the importance of early initiation of CPR by bystanders [6].

These findings indicate that, in addition to bystanders’ ability to perform CPR, educational quality is also crucial to increasing the chance of survival. In several studies, the authors examined the necessary parameters for effective chest compressions and rescue breaths during CPR training with and without feedback (visual/voice) [7,8,9,10,11,12]. Parameters such as the correct hand placement, the right frequency of chest compressions, the correct depth of compressions, adequate chest recoil, and the proper time and volume of rescue breaths influence the outcome of successful resuscitation attempts. Consequently, special attention must be paid to students’ performance by instructors by providing corrective feedback to help ensure consistency of high-quality performance and to increase the trainees’ self-confidence in performing CPR under stressful conditions [13,14,15].

Previous reviews of the literature have provided good evidence supporting the use of CPR feedback/prompt devices during CPR training to improve CPR skill acquisition and retention [16,17,18]. These devices are used frequently in clinical practice and are part of an overall strategy to improve the quality of CPR. Their accuracy in measuring some of the aforementioned parameters, such as the compression depth, accommodate for the calibration needed to adjust the stiffness of the support surface upon which CPR is being performed (e.g., floor/mattress).

Currently, simulation mannequins are manufactured with the ability to provide basic and detailed feedback ability. The former uses an indicator lamp to light up different colors to indicate failure or success in student performance, while the latter shows more detailed and instant information such as exact depth of each individual compression or the pace of the performance. Clinical and practical beliefs among instructors of CPR courses, where this study was conducted, favor the use of instant feedback mannequins. However, there is a lack of adequate studies to demonstrate how students rate CPR learning in these two options [17,18,19,20]. This study, therefore, aimed to evaluate the perceived feasibility of training on a mannequin with basic and detailed instant feedback [21].

## 2. Materials and Methods

### 2.1. Study Design

CPR training began with a theoretical introduction to first aid and CPR. The algorithm for OHCA management in an adult was presented (compliant with the 2015 European Resuscitation Council guidelines) [22]. High-quality CPR was emphasized, and students were familiarized with all the parameters that should be achieved for optimum CPR performance. A demonstration was conducted on an adult Resusci Anne^®^ Skill Reporter^TM^ (New Delhi, India) mannequin from Laerdal Medical with a basic control panel. Afterward, the students practiced CPR on the same mannequin, using the same control panel.

The basic feedback device contained a panel with light-emitting diodes (LEDs) that lit up, informing the students about the following: compression depth level (correct or incorrect), the amount of air during rescue breaths (correct or incorrect), incorrect hand placement for chest compressing (signaled on an image of the chest on the panel), and incorrect airway patency (signaled by the diode placed on the neck shown on the figure indicator).

The feedback received by the basic control panel indicators consisted of lighting up the indicator lamps, where green indicated a well-done activity, while orange and red indicated elements that needed improvement. After connecting the mannequin to the laptop, Skill Reporter^TM^ displayed the exact results of ongoing activities on the monitor screen. Consequently, during chest compression, the student could observe an amplitude graph showing the exact depth of each individual compression and the pace of chest compressions (compressions per minute). During ventilation, the student could observe an amplitude graph showing at what rate air is given and the amount. Students could constantly observe the effects of their actions and incorrect measures at a given moment and try to correct areas of improvement on a regular basis. This detailed feedback gave the opportunity to adjust hand movements to achieve better-quality compressions.

During students’ CPR performance, the instructor observed the following: careful hand placement for resuscitation, the quality of chest compressions performed, and the quality of rescue breaths.

### 2.2. Next Phase

**Figure 1:**
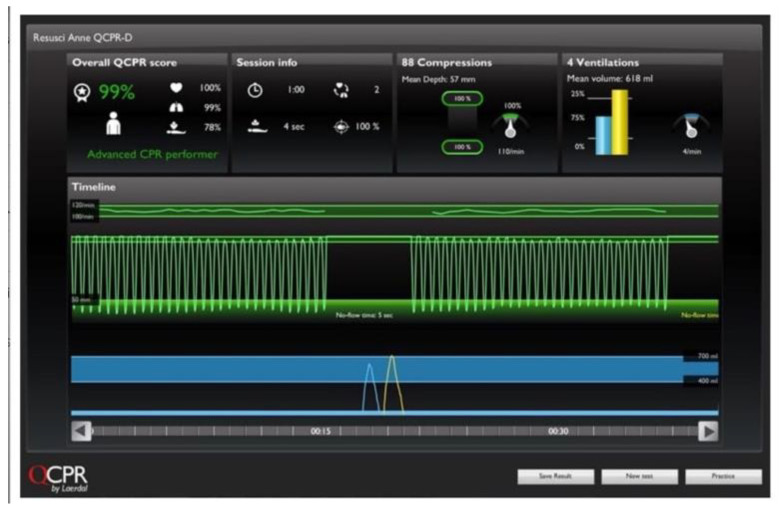
Detailed record of cardiopulmonary resuscitation (CPR) training in progress displayed on the screen.

**Figure 2:**
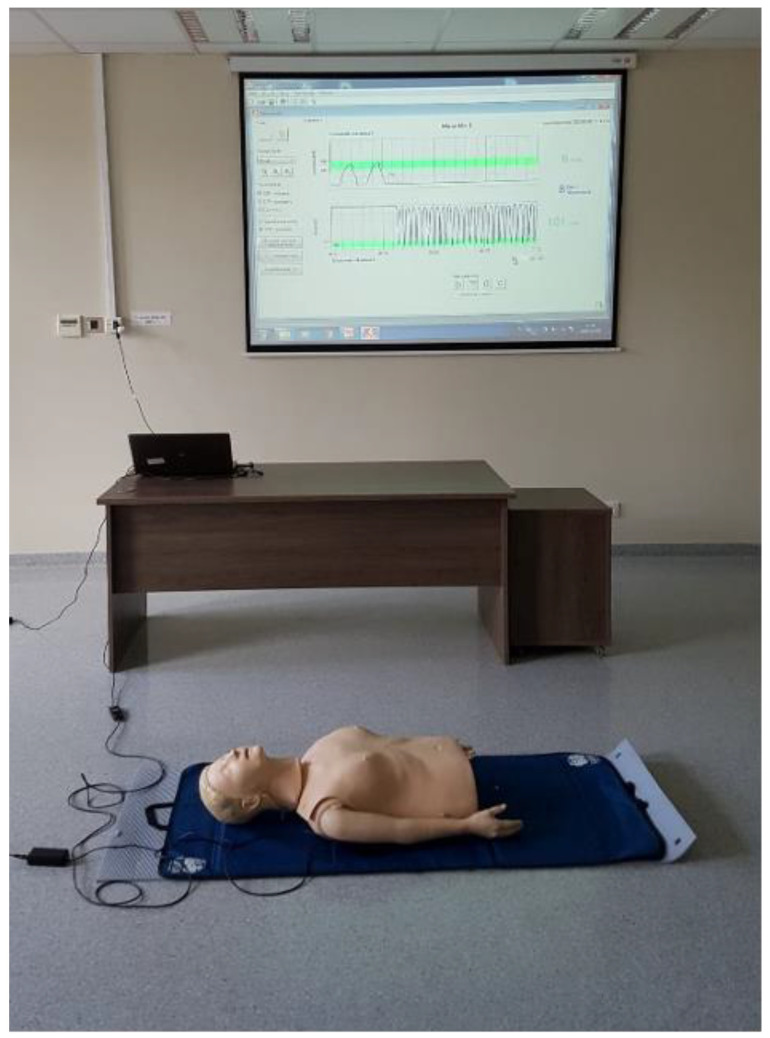
Detailed record of the ongoing CPR displayed on the screen during the research.

In the next class, the same students continued learning CPR with the same mannequin. However, this time, there was an altered form of feedback. The mannequin was connected to a laptop, from which the indicators of resuscitation were displayed on the screen to the class using a projector. The outcome of the resuscitation was displayed synchronously with a millisecond response time. Figure 1 and Figure 2 present a picture of the feedback screen, as seen by the students.

A detailed record presented the following:

- Current and average compression rate,
- The exact number of compressions implemented,
- The ratio of the number of chest compressions to the number of rescue breaths,
- Compression depth given in millimeters,
- Relaxation error,
- Hand position error, indicating the direction of the wrong hand position,
- The airway patency,
- The exact amount of air supplied,
- The speed with which the air is introduced during rescue breaths,
- Resuscitation time.

Students were able to observe their performance and correct the quality of CPR activities on an ongoing basis. Moreover, the instructor conducting the classes observed the students and analyzed their behavior during exercises with the device and gave detailed feedback.

### 2.3. Study Location and Population

The research was conducted from January 2018 to December 2020 at the Medical University of Lublin, Eastern Poland, during first aid classes on basic life support (BLS) in the following faculties: dietetics, cosmetology, physiotherapy, pharmacy, public health, and medical emergency. 

All 709 students receiving training participated in the study (100%). After setting aside incomplete questionnaires, 694 students (98%) qualified for further evaluation, of which 416 were from the Faculty of Health Sciences (dietetics, physiotherapy, medical emergency, and public health) and 278 came from the Faculty of Pharmacy (pharmacy and cosmetology).

### 2.4. Questionnaire

A questionnaire was developed using multiple strategies. First, the authors conducted a literature review to identify the critical dimensions for developing a questionnaire to be used after practical sessions and for the aim of this study. For the review purpose, the following keywords alone or in combination were used: first aid, education, basic life support, cardiopulmonary resuscitation, feedback device, simulation, quality, skill retention, motivation, chest recoil, and mannequins. The acquired data from PUBMED, SCOPUS, and Web of Science were organized, categorized, and mapped to create the questions in the questionnaire. Secondly, a total of nine questions were chosen through the nominal group technique [23] with five students from a local university to evaluate the questionnaire (Appendix A). These participants were later excluded from the study, and their responses were not used in the final analysis. Validity was reviewed on the basis of a combination of logic, relevance, comprehension, legibility, clarity, and usability before the final administration. 

### 2.5. Statistical Analysis

Statistical analysis was performed using SPSS Statistics version 25. A level of α < 0.05 was considered statistically significant. Due to the ordinal and nominal nature of the measurement of variables considered essential for the study, nonparametric tests were used. The median, mean, and standard deviation values, percentage distribution, and mean rank were used to describe the obtained results’ distribution. The mannequin-dependent score measurements were compared using the Wilcoxon test. Glass’s delta expressed the effect size for the calculated difference. The Mann–Whitney U test was used to compare the two groups in terms of the ordinal variable, and the H Kruskal–Wallis test was used to compare more groups. The Mann–Whitney U test was also used as a post hoc test. Its significance level was adjusted using the Bonferroni correction. The effect size for the calculated differences was expressed using Glass’s *r* coefficient and eta-square. Comparing the groups in terms of the qualitative variable was done using the chi-square test, while a *Z* test with Bonferroni correction was used to compare the columns’ proportions. The effect size for the calculated differences was expressed using Cramer’s V coefficient.

### 2.6. Ethics Approval and Consent to Participate

The information included the study’s purpose, the voluntary nature of their participation, strict confidentiality, and secure data storage. The survey was anonymous, and all respondents agreed to participate in the survey. Verbal and informed consent was obtained from participants who completed the paper questionnaire. This study complied with the ethical principles stipulated by Polish law and, thus, was exempt from ethics approval requirements by a named institutional and/or licensing committee.

## 3. Results

The average age of the respondents was 20.89 years, with a standard deviation of 1.54. The youngest respondent was 19 years old, and the oldest was 30 years old. The most frequent age group was 19 years old, accounting for 29.80% of all respondents. The second-largest group was the group of respondents aged 20 (28.00%), and the third-largest group was aged 21 (19.60%). More detailed data on the characteristics of the studied group are presented in Table 1.

**Figure 3:**
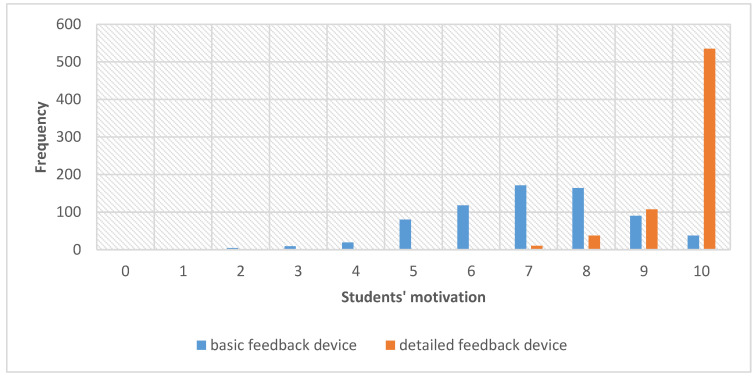
Feedback assessment depending on its type.

Figure 3 presents the distribution of the answers given by the respondents according to the degree of students’ motivation to learn. On an 11-point scale, subjects marked both types of feedback, separately for classes with basic and detailed feedback devices, providing their opinion on their motivation to practice CPR. A closer answer to 10 denoted higher motivation to work with the given feedback, while values close to 0 indicated no such motivation.

The respondents scored the assessed detailed feedback significantly higher than the basic one, as shown by the Wilcoxon test (*Z* = 21.941; *p* < 0.001, *r*_g_ = 0.832). The median score for high-fidelity mannequins was 10 (Me = 10), while the median score for low-fidelity mannequins was 7 (Me = 7). The H Kruskal–Wallis test was used to investigate the relationship between the respondents studying in different faculties and the feedback assessment. The test showed statistically significant yet minute differences between the abovementioned groups of respondents in the assessment of basic feedback (χ^2^ = 27.594; *p* < 0.001; η^2^ = 0.027) and detailed feedback (χ^2^ = 25.175; *p* = 0.001; η^2^ = 0.024). The medians of the results obtained by the groups of respondents and the post hoc tests carried out in pairs to examine the differences in detail are presented in Table 2.

The observed differences were statistically significant in assessing the basic feedback device in the view of respondents from pharmacy and those from public health, dietetics (second and fourth year), and cosmetology.

Respondents studying pharmacy assessed the detailed feedback device statistically higher compared to the respondents studying the combination of emergency medical services and pharmacy.

**Figure 4:**
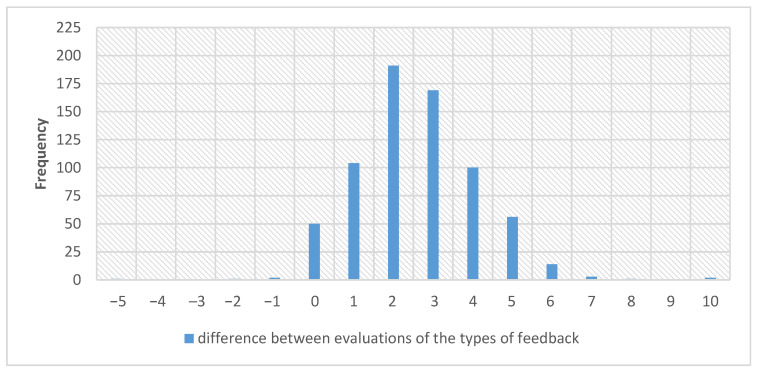
The difference between the high-fidelity mannequin and the low-fidelity mannequin scores.

Figure 4 shows the difference between the high-fidelity mannequin and the low-fidelity mannequin scores. Negative results indicate a better response to the high-fidelity mannequin, and positive results indicate a better response to the low-fidelity one.

The vast majority of respondents, as shown above, assessed detailed feedback higher than the basic one. Only four (0.6%) respondents rated the basic feedback higher than the detailed feedback device. A small number of respondents (*n* = 50; 7.2%) did not notice any difference between the two types of feedback.

Three groups of respondents divided according to the scheme above (lower ratings for detailed feedback, the same assessment for both feedback types, higher ratings for detailed feedback) were compared in terms of response to displaying their CPR outcomes on the projector (in the group) and in terms of self-assessment of their CPR ability. Analysis conducted using the chi-square test showed a statistically significant albeit weak relationship between the assessment of the feedback type and the reaction to displaying respondents’ outcomes on the projector in the presence of the group (χ^2^ = 24.061; *p* < 0.001; V = 0.132) (Table 3). However, no correlation was found between the difference in the assessment of feedback types and the overall self-assessment of CPR skills (Kruskal–Wallis test = 2.489; *p* = 0.288). The median self-assessment of the ability to provide CPR in the group of respondents who rated the basic feedback higher was 3.50 (Me = 3.50), while the median in the group of respondents who assessed the detailed feedback higher and assessed them both at the same level was 4 (Me = 4).

Comparing the proportions of the columns using the *Z* test showed that the respondents who preferred the basic feedback, more often than the respondents preferring the detailed feedback or those without the preference, experienced adverse embarrassment to the presentation of their outcomes on the projector.

The next stage of the analysis was to answer the question of whether the belief about which type of feedback should be used in CPR classes is related to the fear of displaying the subject’s outcomes on the projector in front of the entire group and to the self-assessment of one’s own CPR abilities. The Mann–Whitney U test (U = 12,045.00; *p* = 0.257) and the chi-square test (χ^2^ = 0.404; *p* = 0.816) did not reveal any statistically significant relationships between the abovementioned variables. The median self-assessment of one’s abilities, both in the group of respondents who believed that classes should be conducted with a basic feedback device and in the group of respondents who believed that a detailed feedback device is best suited, was 4 (Me = 4). The distribution of respondents’ responses to displaying their outcomes on the projector is presented in Table 4.

In the group of respondents who believed that it was more reasonable to use a basic feedback device during CPR practice, 5% declared experiencing negative emotions when their actions were displayed on a projector, and 12.5% reported a neutral attitude. In comparison, 82.5% experienced an increase in motivation in such a situation of social exposure. In the group of respondents who believed that it was more appropriate to use the detailed feedback device during CPR classes, 4.13% reported experiencing negative emotions when their actions were displayed on a projector, while 9.79% reported a neutral attitude toward it. In comparison, 86.09% experienced an increase in motivation in this situation.

There was no significant difference in the BLS management algorithm’s knowledge, regardless of the used feedback method. Minor errors in sequencing the procedure or forgetting some activities happened when using either a basic or a more detailed feedback tool. However, a significant difference was observed in students who practiced with a detailed feedback device. The students strived more to achieve high quality concerning both chest compressions and rescue breaths than when working with a basic feedback device.

Moreover, receiving information about mistakes made was better received by students when the information came from the device than from the instructor. Students accepted corrective instruction from the computer program record and found it more objective compared to the instructor. In situations where corrective action was recommended by the instructor, some students interpreted this as instructor bias against the student and viewed this as unjustified criticism. More than once, students tried to contest a negative comment, questioning and failing to accept it, or requesting the instructor to give a higher grade by disputing error occurrence. Conversely, the device’s result was not subject to discussion on the student’s part; it was accepted, and the students understood improvement was required.

## 4. Discussion

The World Health Organization aims to empower communities during disasters and emergencies [24,25,26,27]. First aid and CPR skill training contribute to community preparedness and resilience and should be widely available [28]. There is scientific evidence that introducing lay BLS courses improved survival rates 30 days and 1 year after sudden cardiac arrest [29,30]. Given the positive response from student health professionals, to improve skill acquisition and retention in BLS courses for health and laypeople, detailed feedback should be provided during all CPR training. In particular, high-fidelity device training that provides instant feedback should be available to students pursuing health professions in terms of training and careers, as well as laypeople who are obliged to provide first aid, e.g., teachers, medical guardians, security guards, and police officers. From a cognitive perspective, learning during training signifies an individual’s capacity to obtain and reflect on external information to apply it in real-life scenarios [31,32,33,34,35]. To optimize feasibility, there needs to be a constructive alignment between learning goals and learning activities [36]. All students have different ways of learning. As Hattie discovered, students have additional attributes, prior knowledge, motivations, and intentions to participate in learning, thus leading to significant variance [37].

According to this study, students learning with mannequins that provided instant and accurate feedback were more engaged and motivated to have the best-quality CPR result. These results were statistically significant regardless of the participants’ field of study, with the vast majority of students highlighting that their CPR practice should be based on mannequins providing detailed feedback (94.2%).

Most often, students had prior experience with basic mannequins that made a sound or showed a light when pressed, with a basic control panel and no feedback. As a result, their actions were often rated very high or entirely correct, and the students were, thus, convinced that CPR was very simple to perform. However, after using the mannequin with detailed feedback, the students realized that they did not achieve the recommended depth of compressions, usually compressed too quickly, had problems with proper airway patency, and gave far too little or too much air during rescue breaths. In accordance with other studies receiving detailed feedback from the device, this enabled them to improve their CPR results to as high as 100% [38,39,40]. Students practiced on the mannequin willingly; students (mostly male students) asked to be allowed to test/retest their abilities many times. This willingness to practice allowed the students to measure the time in which their activities resulted in effective resuscitation until the quality of their actions clearly decreased. On the first day of the class, when the exercises were performed on the mannequin with a basic control panel, the students performed CPR less willingly, without significant engagement. Eshel et al. reached similar conclusions when comparing the CPR quality performed on two student groups [41]. One class (the control group) was taught using standard mannequin-based CPR models. The second class (the intervention group) was taught similarly but with real-time CPR quality feedback. In multiple regression analysis, the real-time feedback group results were significantly better than the control group in all baseline scores, adjusted to the participant’s age, gender, and body mass index characteristics [35]. Another study similar to the present report was conducted by Lu et al., in which the authors verified whether a smartwatch with real-time feedback could improve the quality of CPR performed by healthcare professionals [42]. It showed that, without real-time feedback, chest compressions were usually too fast and too shallow, and that CPR quality could be improved with a smartwatch that provides real-time feedback [43]. Tanaka et al. conducted a randomized, controlled trial to compare standard CPR training (control) and QCPR Classroom (intervention) amongst 642 Japanese students over 15 years of age. As in this study, QCPR Classroom participants could see their CPR results on a large screen, while the control group only received subjective instructor feedback. QCPR Classroom was found to help students obtain high-quality CPR training, especially in terms of correct compression depth and full recoil [44]. Other research studying the influence of received feedback on CPR quality [45,46] reported similar conclusions.

During the severe acute respiratory syndrome coronavirus 2 (SARS-CoV-2; COVID-19) pandemic, the provision of mouth-to-mouth rescue breaths by lay people is not recommended due to the risk of transmission of infection [47]. Therefore, the focus should be on high-quality chest compressions [48]. A study by Stiell et al. proved the importance of chest compressions quality during CPR. A study of patients with prehospital sudden cardiac arrest demonstrated that increased compression depth in CPR is strongly associated with better survival [49]. Maximum survival was in the depth range of 40.3 to 55.3 mm (peak, 45.6 mm) [50]. However, in this study, approximately 20% of participants showed a lack of full chest expansion during CPR, claiming that that no one had previously informed them about the significance and possibility of this error. As noted in the European Resuscitation Council (ERC) 2015 guidelines, it is often found that the rescuer’s hands rest on the chest during CPR such that the return to its original shape is incomplete [51], while a full chest recoil after each compression results in a better return of venous blood to the chest, which may improve CPR effectiveness [52].

In this study, the authors recognize that the students better perceived receiving information about mistakes when the information came from the device than from the instructor. Another argument in favor of introducing accurate feedback devices can be derived from the exciting results of the research by Hansen et al., who investigated whether BLS instructors correctly assess medical students’ CPR skills [52]. They found that certified BLS instructors rated the CPR abilities of participants poorly. Of the 90 CPR assessments conducted by 16 pairs of instructors, 90% passed the exam (81 students), while the mannequin pass rate was only 2% [52]. Other authors have also come to similar conclusions [53,54,55,56,57].

Another interesting result of this study was the outcome of participants’ self-assessment of CPR skills. All students assessed themselves very similarly, regardless of whether they were students of emergency medical services, whose profession would involve performing medical rescue activities, or students of other faculties, e.g., cosmetology or public health. Students did not rate themselves highly; most of them rated their skills as 4 on a five-point Likert scale, indicating a high awareness of their deficits and the need for regular practice and recall of CPR procedures. It can be concluded, after the class using a mannequin with detailed feedback, that students were aware that not everything was performed correctly. They observed that it was not easy to maintain the correct pace of compressions or maintain an appropriate depth; hence, they approached self-assessment critically and objectively. According to their assessment, they somehow demonstrated that the highest rating (5) requires regular exercise. The only similar study that we could refer to when comparing our results was the study by Abolfotouh et al., who verified, among other things, the importance of mental attitude in respondents toward CPR [58]. A positive attitude was significantly more often shown, in statistical terms, by participants who recently completed BLS training, had a higher number of previous BLS training sessions, and/or had earlier exposure to cardiac arrest, which means that, with more experience, self-confidence, and self-esteem, one’s abilities increase.

## 5. Limitations

This study has some limitations. This was a convenience sample from one university. Accordingly, a lack of medical students from the medical faculty participated because the emergency department does not conduct CPR classes for them. This study could be performed with two groups (control and study) to further evaluate the differences. The outcome of this study indicates a need for further controlled studies with a larger sample. This study may also initiate a discussion on this subject and the perspective of broader research in this area. The experience gained from this study can form the basis for planned future research, comparing training methods in other countries globally, thereby providing a wider standardization of the research tool used.

## 6. Conclusions

This study found that detailed feedback provided via training mannequins increases participants’ motivation to learn and improve their CPR proficiency. Devices that provide detailed feedback during BLS courses may contribute to improving overall CPR quality, increasing the chances of survival in cases of OHCA. Providing detailed feedback concerning CPR provider performance in the presence of other students may be an effective motivator to improve or correct technique.

## Figures and Tables

**Table 1:** Characteristics of the researched group.

Faculty	*n*	%	M (%)	Female	Male
Dietetics II year	136	20	21.33	124	12
Dietetics IV year	30	4	23.12	29	1
Pharmacy I year	105	15	20.08	87	18
Pharmacy IV year	41	6	23.04	29	12
Physiotherapy I year	118	17	19.48	76	42
Cosmetology I year	132	19	19.19	132	0
Public Health II year	51	7	20.29	43	8
Medical Rescue I year	81	12	20.67	26	55
**Total**	**694**	**100**	**20.89**	**552**	**157**

**Table 2:** Mannequin assessment depending on the respondents’ group.

Feedback Type	Faculty	M	*n*	Me	Pharmacy IV Year	Dietetics IV Year	Dietetics II Year	Medical Rescue I Year	Physiotherapy I Year	Public Health II Year	Pharmacy I Year
basic	Cosmetology I year	372.33	132	7	2404.5	1761.0	8152.5	5286.5	6328.5	3154.5	5147.5 *
	Pharmacy IV year	335.59	41	7		469.0	2529.5	1475.5	2191.0	853.0	1785.5
	Dietetics IV year	412.63	30	8			1746.0	1113.0	1225.0	730.0	962.0 *
	Dietetics II year	365.82	136	7				5340.0	6607.5	3165.5	5336.5 *
	Medical Rescue I year	372.43	81	7					3954.0	1962.5	3265.0
	Physiotherapy I year	306.33	118	7						2242.5	5813.0
	Public Health II year	395.00	51	7							1796.0 **
	Pharmacy I year	282.58	105	7							
detailed	Cosmetology I year	333.42	132	10	2266.0	1782.5	8402.5	4994.0	7374.5	2985.5	5844.5
	Pharmacy IV year	278.71	41	10		461.5	2160.5	1513.0	1905.5	766.5	1493.0 **
	Dietetics IV year	367.92	30	10			1960.5	1023.5	1685.0	755.5	1490.0
	Dietetics II year	355.14	136	10				4811.0	7942.0	3290.5	6456.5
	Medical Rescue I year	311.65	81	10					4225.5	1710.0	3351.0 *
	Physiotherapy I year	351.60	118	10						2826.0	5548.5
	Public Health II year	372.41	51	10							2563.0
	Pharmacy I year	387.27	105	10							

* *p* < 0.05; ** *p* < 0.01; M—average rank; *n*—number; Me—median.

**Table 3:** Distribution of the feedback type by the respondents and the reaction to displaying their outcomes on a projector in the presence of their group.

Students’ Reaction to Displaying Their Outcomes on a Projector	*n*	Feedback Preferences		
		Basic Feedback	No Preference	Detailed Feedback
Adverse—embarrassment	*n*	2 _a_	0 _b_	27 _b_
	%	50.00%	0.00%	4.22%
Positive—motivation increase	*n*	2 _a_	43 _a_	551 _a_
	%	50.00%	86.00%	86.09%
Neutral	*n*	0 _a_	7 _a_	62 _a_
	%	0.00%	14.00%	9.69%

Each subscript letter represents a subset of the feedback preference category whose column proportions do not differ significantly (by 0.05).

**Table 4:** Distribution of basic and detailed feedback devices in conducting CPR classes and the reaction to displaying their work on a projector in the presence of a group.

Students’ Reaction to Displaying Their Outcomes on a Projector	*n*	The Belief in the Usefulness of the Given Feedback	
		Highest Usefulness of Basic Feedback Device	Highest Usefulness of Detailed Feedback Device
Adverse—embarrassment	*n*	2 _a_	27 _a_
	%	5.00%	4.13%
Positive—motivation increase	*n*	33 _a_	563 _a_
	%	82.50%	86.09%
Neutral	*n*	5 _a_	64 _a_
	%	12.50%	9.79%

Each subscript letter represents a subset of the feedback preference category whose column proportions do not differ significantly (by 0.05).

## Data Availability

Datasets used and analyzed during the current study are available from the corresponding author on reasonable request.

## Appendix A

### The Questionnaire

Please read the questionnaire carefully and fill it in. When answering, please tick the appropriate boxes. The survey is completely anonymous. Your responses will only be used for statistical summaries.

1. **How would you rate your motivation to learn CPR on a simple mannequin that gives green- or red-light feedback (good/bad)? Did you have a strong desire to improve your skills while practicing on this mannequin?**

I have no motivation 1 2 3 4 5 6 7 8 9 10 I have motivation

2. **How do you evaluate your motivation to learn CPR on a high-fidelity mannequin that provides detailed projector feedback on a large screen? While learning on this mannequin, did you have a strong desire to improve your skills?**

I have no motivation 1 2 3 4 5 6 7 8 9 10 I have motivation

3. **Did it bother you that the effect of your work was displayed on the projector screen or did it motivate you to work better?**

- It bothered me, it intimidated me
- It motivated me to work better, to improve my skills
- It did not bother me or motivate me
- Other

4. **How would you rate your CPR skills overall?**

Very low 1 2 3 4 5 6 7 8 9 10 Very Good

5. **Do you think simple low-fidelity mannequins are sufficient to teach first aid as part of your field of study or should classes be taught on high-fidelity mannequins with advanced feedback?**

- Simple low-fidelity phantoms are sufficient
- Classes should be conducted on high-fidelity phantoms with detailed feedback

6. **Faculty**

- Dietetics
- Physiotherapy
- Cosmetology
- Medical Rescue
- Other

7. **Year of study**

- First
- Second
- Third

8. **Gender**

- Male
- Female

9. **Age**

- 19
- 20
- 21
- 22
- 23
- 24
- Other
